# Increased Antibody Response to Fucosylated Oligosaccharides and Fucose-Carrying *Bacteroides* Species in Crohn’s Disease

**DOI:** 10.3389/fmicb.2020.01553

**Published:** 2020-07-14

**Authors:** Katharina Kappler, Yi Lasanajak, David F. Smith, Lennart Opitz, Thierry Hennet

**Affiliations:** ^1^Institute of Physiology, University of Zurich, Zurich, Switzerland; ^2^Department of Biochemistry, Emory Comprehensive Glycomics Core, Emory University School of Medicine, Atlanta, GA, United States; ^3^Functional Genomics Center Zurich, ETH Zurich/University of Zurich, Zurich, Switzerland

**Keywords:** IBD, oligosaccharide, array, microbiota, *Bacteroides*, fucose

## Abstract

Inflammatory bowel disease is associated with intestinal dysbiosis and with elevated antibody production toward microbial epitopes. The underlying processes linking the gut microbiota with inflammation are still unclear. Considering the constant induction of antibodies by gut microbial glycans, the aim of this study was to address whether the repertoire of carbohydrate-specific antibodies is altered in Crohn’s disease or ulcerative colitis. IgG and IgM reactivities to oligosaccharides representative of mucosal glycans were tested in blood serum from 20 healthy control subjects, 17 ulcerative colitis patients, and 23 Crohn’s disease patients using glycan arrays. An increased IgG and IgM reactivity toward fucosylated oligosaccharides was detected in Crohn’s disease but not in ulcerative colitis. To address the antibody reactivity to the gut microbiota, IgG binding to members of a complex intestinal microbiota was measured and observed to be increased in sera of patients with Crohn’s disease. Based on the elevated reactivity to fucosylated oligosaccharides, gut bacteria were tested for recognition by the fucose-binding *Aleuria aurantia* lectin. *Bacteroides stercoris* was detected in IgG- and lectin-positive fractions and reactivity of *A. aurantia* lectin was demonstrated for additional *Bacteroides* species. IgG reactivity to these *Bacteroides* species was significantly increased in inflammatory bowel disease patients, indicating that the increased reactivity to fucosylated oligosaccharides detected in Crohn’s disease may be induced by fucose-carrying intestinal bacteria. Enhanced antibody response to fucosylated epitopes may have systemic effects by altering the binding of circulating antibodies to endogenous glycoproteins.

## Introduction

Inflammatory bowel disease (IBD) encompasses a group of chronic inflammatory diseases of the gastrointestinal tract including the two major types of ulcerative colitis (UC) and Crohn’s disease (CD). IBD is a multifactorial disease associated with abnormal mucosal immune responses combined with altered function of the intestinal barrier and genetic and environmental factors (Ramos and Papadakis, 2019). Despite differences between UC and CD, several features are common to both conditions. UC mainly affects the mucosal layer of the colon, whereas CD is characterized by alterations of the whole gastrointestinal bowel wall (Waugh et al., 2013). Intestinal dysbiosis is frequent in UC and CD (Nishida et al., 2018). Decreased microbial diversity has been reported for both diseases, but is often more pronounced in CD (Ott et al., 2004; Manichanh et al., 2006). Despite controversial results on the increase or decrease of specific taxa, studies consistently reported a lower abundance of Firmicutes in IBD (Manichanh et al., 2006; Frank et al., 2007; Nishida et al., 2018). Furthermore, IBD is associated with an increase of potentially inflammatory bacteria, such as adherent-invasive *Escherichia coli* (Martin et al., 2004; Nishida et al., 2018), and a decrease of bacteria with anti-inflammatory characteristics, such as *Faecalibacterium prausnitzii* (Fujimoto et al., 2013; Machiels et al., 2014; Takahashi et al., 2016). A higher abundance of Bacteroidetes and Proteobacteria, including *Enterobacteriaceae* (Seksik et al., 2003; Gophna et al., 2006), has been reported, even though there are also studies that showed a decrease of Bacteroidetes (Frank et al., 2007).

IBD is associated with elevated titers of antibodies targeting microbial epitopes (Mitsuyama et al., 2016). These antibodies include a panel of carbohydrate-reactive antibodies, such as anti-laminaribioside, anti-mannobioside, anti-chitobioside, anti-laminarin and anti-chitin (Dotan et al., 2006; Rieder et al., 2010; Kaul et al., 2012; Paul et al., 2015). Exposure to surface glycans of intestinal bacteria triggers the production of carbohydrate-specific antibodies (Springer et al., 1961; Springer and Horton, 1969; Macher and Galili, 2008; Yilmaz et al., 2014; Bello-Gil et al., 2019). Some bacterial and mammalian glycans share antigenic properties, for example AB0 blood group antigens, which stimulate the production of AB0-specific antibodies during the first months of life after microbial colonization of the gastrointestinal tract (Springer and Horton, 1969; Dean, 2005). Specific bacteria mimic host glycan structures to evade recognition by the immune system (Moran et al., 1996; Comstock and Kasper, 2006). Antigen mimicry may lead to the formation of cross-reacting antibodies that recognize structurally related host glycans. Antibodies against the lipooligosaccharide coat of *Campylobacter jejuni* can react with the structurally related ganglioside GM1 and trigger Guillain-Barré syndrome (Yuki et al., 2004). In a similar manner, antibodies to bacterial glycans can recognize similar epitopes expressed on the intestinal mucosa, thereby possibly contributing to a local inflammatory response.

Considering the dysbiosis associated with IBD, changes in the intestinal microbiota may lead to the emergence of novel antibodies cross-reacting with host intestinal glycans. To address whether the repertoire of carbohydrate-specific antibodies is altered in IBD, and whether changes in antibody profiles can be linked to the expansion of specific bacterial taxa, we investigated the reactivity of serum antibodies to mucosal glycans in IBD patients using oligosaccharide arrays.

## Results

### Increased Serum Antibody Response to Oligosaccharides in Crohn’s Disease

**FIGURE 1:**
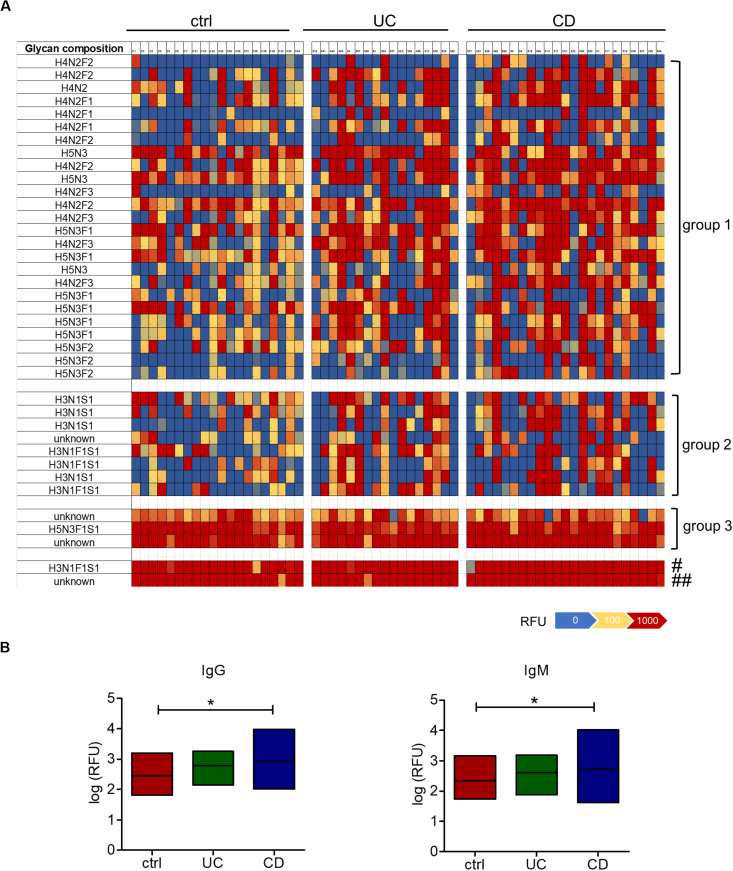
Increased serum IgM and IgG reactivity to oligosaccharides in CD patients. **(A)** Heatmap of the IgG reactivity to oligosaccharide structures for blood serum from healthy controls (*n* = 20), UC (*n* = 17) and CD (*n* = 23) patients, based on the fluorescence intensities (RFU) measured by glycan array analysis. Shown are oligosaccharides or groups of oligosaccharides with high IgG reactivities in the CD patients group (group 1, group 2) or in all groups (group 3 and two single structures, marked with # and ##). The composition of oligosaccharides is indicated by the number of different monosaccharides (hexose, H; HexNAc, N; fucose, F; sialic acid, S). **(B)** IgG and IgM reactivities shown as median log (RFU) of all 220 oligosaccharides tested on the glycan array. *p-value ≥ 0.05, determined using one-way ANOVA and Bonferroni’s *post hoc* test with selected pairs for ctrl vs UC and ctrl vs CD.

To analyze the occurrence and diversity of oligosaccharide-specific antibodies, we determined the reactivity of serum IgM and IgG to arrays displaying 220 distinct human milk oligosaccharide structures (Yu et al., 2014). Human milk oligosaccharides share structural similarities and common epitopes with mucosal glycans, making them suitable for the analysis of antibodies to mucosal glycans (Marcobal et al., 2011; Koropatkin et al., 2012). For these structures only the composition in terms of the numbers of monosaccharides was known and abbreviated by the number of hexoses (H), N-acetyl-hexosamine (N), fucose (F) and N-acetylneuraminic acid as sialic acid (S) (Supplementary Table S1). We compared 17 sera from UC and 23 sera from CD patients with 20 sera from healthy control subjects. Heatmaps of the whole data set showed that patterns of IgG and IgM reactivity diverged between individual sera (Supplementary Figure S1), but consistent changes in subgroups of oligosaccharides were observed. Strikingly high antibody reactivities in the patients were observed for two groups of oligosaccharides. Group 1 comprised various undecorated and fucosylated H4N2 and H5N3 core structures, whereas group 2 consisted of different oligosaccharides with the composition H3N1F1S1 and H3N1S1 (Figure 1A, Supplementary Figure S1). In addition, we observed very high reactivities not only in patients’ samples but also in all control samples for a third group and two additional structures (Figure 1A, Supplementary Figure S1). One of the two single oligosaccharides represented H3N1F1S1 and one structure of group 3 consisted of H5N3F1S1, whereas the compositions of the other three oligosaccharides were unknown. Overall, the reactivity of IgG and IgM toward oligosaccharides was increased in CD sera, whereas IgG and IgM responses remained unchanged in UC sera (Figure 1B). The patterns of oligosaccharides recognized by an individual serum was similar between IgG and IgM (Supplementary Figure S2). IgG and IgM reactivities correlated significantly for each serum sample tested. For 39 out of 40 samples the mean correlation coefficient ranged between 0.67 and 0.98 (Supplementary Table S2). Because of this similarity, we focused our subsequent analyses on IgG reactivities.

**FIGURE 2:**
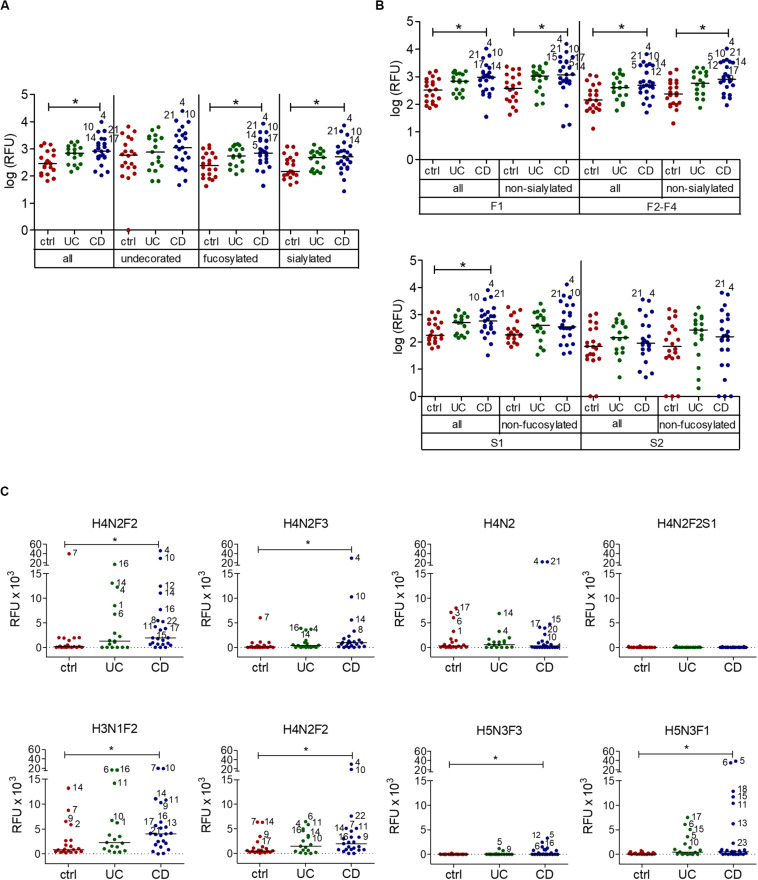
Serum IgG reactivity to fucosylated oligosaccharides in CD patients. **(A)** Median IgG reactivities to subgroups of oligosaccharides on the array shown as log (RFU). **(B)** Analysis of subgroups of fucosylated and sialylated oligosaccharides (mono-fucosylated, F1; di- to tetra-fucosylated, F2-F4; mono-sialylated, S1; di-sialylated, S2). **(C)** Median IgG reactivity to single exemplary fucosylated oligosaccharides and correspondingly undecorated and sialylated oligosaccharides, shown as RFU. The composition of oligosaccharides is indicated by the number of different monosaccharides (hexose, H; HexNAc, N; fucose, F; sialic acid, S). All panels include 20 controls, 17 UC and 23 CD samples. * *p*-value ≥ 0.05, determined using one-way ANOVA and Bonferroni’s *post hoc* test for panel **(A)** and **(B)** or Kruskal-Wallis test and Dunn’s *post hoc* test for panel **(C)**, both with selected pairs of ctrl vs UC and ctrl vs CD.

When comparing the IgG reactivity to different oligosaccharide structures on the array, we analyzed the IgG binding to oligosaccharides carrying fucose and sialic acid, which are typical epitopes found on intestinal glycans (Robbe et al., 2004). IgG response to oligosaccharides containing fucose or sialic acid was increased in CD sera, whereas recognition of undecorated oligosaccharides lacking fucose or sialic acid residues remained unchanged (Figure 2A). The reactivity of UC sera to different subsets of oligosaccharides also remained unchanged compared with healthy control sera. Closer analysis of the subgroups of fucosylated and sialylated oligosaccharides indicated that the change of IgG reactivity to oligosaccharides in CD mainly reflected an increased reactivity toward fucosylated but non-sialylated oligosaccharides. The group of oligosaccharides carrying two to four fucose residues (F2–F4) showed the largest difference in IgG reactivity between healthy controls and CD patients (Figure 2B). Again, IgG reactivity from UC sera toward subtypes of oligosaccharides remained unchanged. Hierarchical cluster analysis of the same data confirmed that the majority of control samples did not belong to the group of samples with high IgG reactivity to fucosylated oligosaccharides, in which CD samples and UC samples were frequent (Supplementary Figure S3). The contribution of fucosylated oligosaccharides to the elevated IgG reactivity to oligosaccharides was confirmed by calculating the fold change of IgG reactivity for sample groups (ctrl vs UC, ctrl vs CD) for every single oligosaccharide. Setting a threshold difference of log-fold change (logFC) larger than two, oligosaccharides associated with increased IgG reactivity mainly belonged to fucosylated oligosaccharides, whereas all oligosaccharides with decreased IgG reactivity were sialylated (Table 1).

**TABLE 1:** Oligosaccharides with altered serum IgG reactivity in IBD patients.

Increased IgG reactivity in CD			Increased IgG reactivity in UC		
Composition	logFC	*P*-value	Composition	logFC	*P*-value
H3N1S1	4.19	0.01	H4N2F3	4.40	0.00
H4N2F3	4.02	0.00	H3N1F1S1	3.70	0.02
H6N4F4/H7N5F2	3.75	0.00	H3N1F1 (LNnFP V)	3.33	0.02
H5N3F3	3.67	0.00	H5N3F2	3.22	0.01
H4N2F2	3.67	0.02	H4N2F2	3.22	0.05
H4N2F3	3.49	0.01	H6N4F3	3.10	0.04
H4N2F2S1	3.44	0.01	H5N3F2	2.92	0.03
H7N5F3/H8N6F2	3.39	0.01	H4N2F1S1	2.78	0.03
H6N4F2/H7N5F1	3.34	0.02			
H7N5	3.20	0.01			
H4N2F1S1	3.17	0.01			
H4N2F1S1	3.10	0.05			
H5N3F1	3.07	0.03			
H4N2F3	2.92	0.02			
unknown	2.82	0.01			
H5N3F3	2.81	0.05			
H5N3F1S1	2.78	0.03			
H4N2F3	2.76	0.04			
H4N2F2	2.74	0.03			
H4N2F2	2.70	0.05			
H3N1F2	2.55	0.05			
H5N3F2	2.54	0.04			
H9N7F4	2.44	0.05			
H10N8F4	2.36	0.04			
H2F1 (3-FL)	2.14	0.05			
H4N2F2S1	2.10	0.03			

**Decreased IgG reactivity in CD**			**Decreased IgG reactivity in UC**		
**Composition**	**logFC**	***P*-value**	**Composition**	**logFC**	***P*-value**

H5N3F3S1/H6N4F2S1	2.66	0.02	H5N3F1S1	3.91	0.00
H5N3F1S1	2.54	0.03	H5N3F1S1	3.07	0.01
H3N1S1 (LSTb)	2.51	0.03	H5N3F2S1	3.05	0.01
H6N4S1	2.11	0.02	H5N3F2S2	2.83	0.03
H5N3F1S1	2.03	0.04	H6N4S1	2.72	0.01
			H6N4F1S1	2.46	0.00

*H, hexose; N, N-acetyl-hexosamine; F, fucose; S, sialic acid; 3-FL, 3-Fucosyllactose; LNFP V, Lacto-N-fucopentaose V; LNnFP V, Lacto-N-neofucopentaose V; LNnT, Lacto-N-neotetraose; LST b, Sialyllacto-N-tetraose b.*

IgG reactivity to fucosylated oligosaccharides was higher in CD patients compared with healthy controls. By contrast, IgG reactivity to undecorated and sialylated oligosaccharides was similar between IBD patients and healthy controls (Figure 2C). Interestingly, the presence of sialic acid strongly lowered antibody reactivity, as shown for H4N2F2S1 in comparison to related fucosylated and non-sialylated oligosaccharides, such as H4N2F2 and H4N2F3 (Figure 2C). In general, sialylation was associated with decreased IgG reactivity to oligosaccharides in IBD sera (Table 1), even though one specific sialylated and non-fucosylated oligosaccharide with the composition H3N1S1 was also among the structures associated with an IgG reactivity in IBD sera (Table 1).

### Increased IgG Reactivity to the Intestinal Microbiota in CD

**FIGURE 3:**
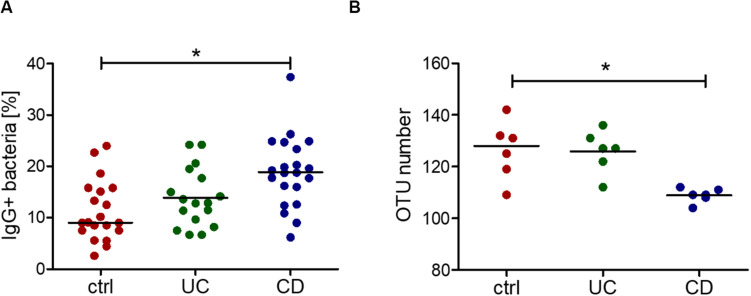
Increased IgG reactivity and decreased IgG repertoire to commensal bacteria in the blood serum of CD patients. **(A)** Percentage of IgG-positive cells of an intestinal complex microbiota, determined by flow cytometry, as the result of three independent experiments (different batches of bacterial culture and different serum samples, *n* = 20 for ctrl, *n* = 17 for UC, *n* = 21 for CD). **(B)** Alpha-diversity, shown as the number of different OTUs within one sample, grouped by control, UC and CD, resulting from sorting and 16S sequencing of IgG-bound bacteria of 6 samples per group. *p-value ≥ 0.05, determined using one-way ANOVA and Bonferroni’s *post hoc* test with selected pairs for ctrl vs UC and ctrl vs CD.

**FIGURE 4:**
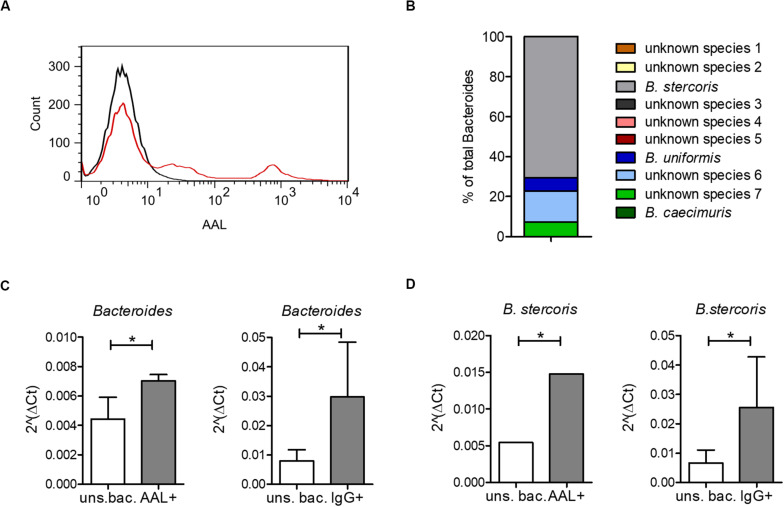
*Bacteroides* recognition by serum IgG and by the fucose-binding lectin AAL. **(A)** AAL-bound bacteria from a healthy human microbiota culture (red) compared with unstained bacteria from the same microbiota sample (black). **(B)** All OTUs identified as *Bacteroides* by 16S sequencing of AAL-bound bacteria as percentage of total bacteria. **(C)** Real-time PCR using specific primers for *Bacteroides*, shown as median ± standard deviation for AAL-bound (AAL+) (*n* = 3) and unsorted bacteria (uns. bac.) (*n* = 3) and for IgG-bound (IgG+) (*n* = 36) and corresponding unsorted bacteria (uns. bac.) (*n* = 4). **(D)** Real-time PCR using specific primers for *B. stercoris*, shown as median ± standard deviation for AAL-bound (AAL+) (*n* = 3) and unsorted bacteria (uns. bac.) (*n* = 3) and for IgG-bound (IgG+) (*n* = 36) and corresponding unsorted bacteria (uns. bac.) (*n* = 4). *p-value ≥ 0.05, determined by student’s *t*-test.

Increased IgG response to commensal bacteria in IBD has been reported not only for mucosal (Macpherson et al., 1996) but also for systemic antibodies (Adams et al., 2008; Haas et al., 2011; Harmsen et al., 2012; Castro-Dopico et al., 2019). Using flow cytometry, we indeed detected increased IgG binding to bacteria from a healthy intestinal microbiota in sera from CD patients compared with healthy controls (Figure 3A). The bacteria bound by serum IgG were isolated and identified by 16S rRNA sequencing for representative six healthy controls, six CD and six UC patients (samples are marked in Supplementary Figure S1). This analysis revealed a decreased alpha-diversity in the CD group but not in UC (Figure 3B), indicating a restricted serum IgG repertoire toward bacterial antigens in CD. Because of the increased reactivity of IgG to fucosylated oligosaccharides observed in CD sera, we analyzed the composition of the bacteria in the healthy human intestinal microbiota that are recognized by the fucose-binding *Aleuria aurantia lectin* (AAL) (Romano et al., 2011). A fraction representing 20% of the complex bacterial mixture was positive for AAL binding (Figure 4A). The AAL-positive fraction was isolated by cell sorting and bacteria were identified by 16S rRNA sequencing. At the genus level, taxa enriched at least 1.5-fold included *Peptinophilus, Paenibacillus, Agathobacter, Anaerostipes, Escherichia, Enterococcus, Eubacterium, Holdemania, Slackia, Proteus, Ruminococcus, Parabacteroides, Ruthenibacterium, Lachnospira, Bacteroides, Murdochiella, Succiniclaticum, Frisingicoccus*, and *Bifidobacterium*. In addition, several unknown genera belonging to the families of *Lachnospiraceae*, *Ruminococcaceae*, and *Muribaculaceae* were also identified (Supplementary Figure S4). The comparison of the bacterial taxa positive for AAL binding and those recognized by IgG from CD sera pointed to specific taxonomical groups and single species found in both fractions. Among the taxa enriched more than 1.5-fold in both AAL-positive and IgG-positive fractions, we focused on bacteria preferentially enriched after capture with IgG from CD sera against IgG from healthy control sera. At the genus level, nine taxa, including *Bacteroides, Slackia, Agathobacter, Enterococcus, Escherichia, Peptoniphilus, Frisingicoccus*, and two unknown taxa, met this criterion. *Bacteroides* was the only taxa that included more than a single species (Figure 4B). Enrichment of *Bacteroides* in the AAL-positive and IgG-positive fractions of CD sera was confirmed by real-time PCR (Figure 4C). *Bacteroides stercoris* was the major species representing *Bacteroides* enrichment in the AAL-positive and IgG-positive samples (Figure 4D). Notably, an increased abundance of *B. stercoris* in fecal samples of CD patients has been previously reported (Walters et al., 2014).

### Bacteroides Fucosylation and Recognition by IgG in CD Patients

**FIGURE 5:**
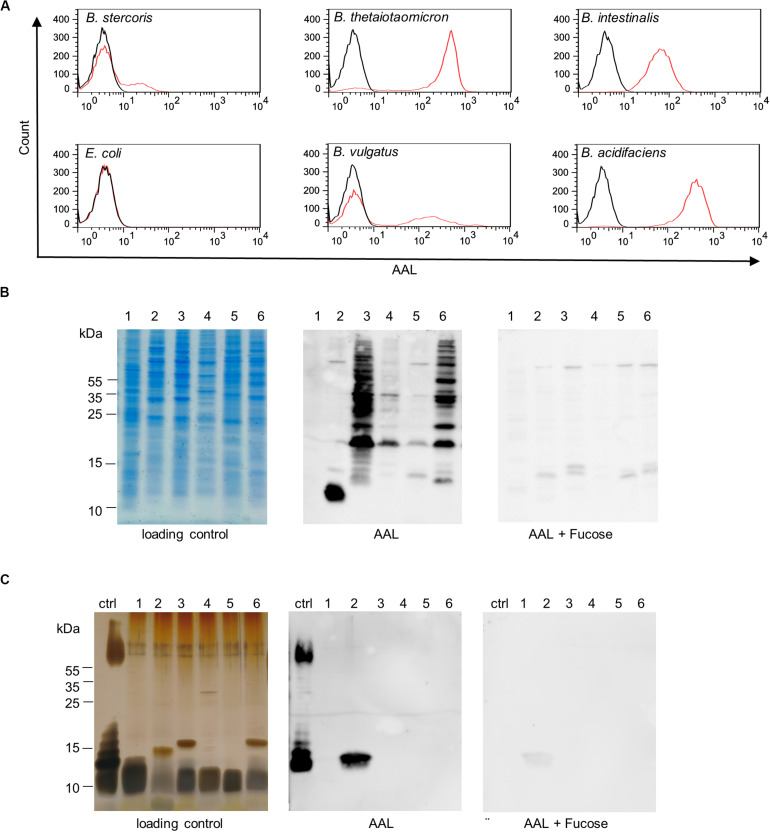
*Bacteroides* surface glycans recognized by fucose-specific lectin AAL. **(A)** Flow cytometry histograms of *Bacteroides* species and *E. coli* K12 stained with fluorescein-AAL (red) compared with unstained bacteria (black). **(B)** Analysis of bacterial cell lysates by SDS-PAGE and Coomassie (loading control) and by lectin blotting (AAL). The specificity of the AAL signal was confirmed using competing soluble L-fucose (AAL + Fucose). **(C)** Analysis of isolated LPS by SDS-PAGE and silver stain (loading control) and by lectin blotting (AAL). The specificity of AAL was tested with the inhibiting sugar L-fucose (AAL + Fucose). Ctrl, *E. coli* O127:B8; 1, *E. coli* K12; 2, *B. intestinalis*; 3, *B. thetaiotaomicron;* 4, *B. vulgatus*; 5, *B. stercoris*; 6, *B. acidifaciens*.

Bacteroidetes is a major phylum of the human intestinal microbiota and several *Bacteroides* species have been found to be enriched in IBD (Swidsinski et al., 2005; Gevers et al., 2014). The abundance of surface fucosylation on intestinal *Bacteroides* species was assessed by analyzing AAL binding to bacteria using flow cytometry. Fucosylation was confirmed for *B. stercoris* and further demonstrated for all additional *Bacteroides* species tested, namely *B. vulgatus*, *B. intestinalis, B. acidifaciens*, and *B. thetaiotaomicron* (Figure 5A). As expected, *E. coli* K12 that lacks fucosylated epitopes and was included as a negative control remained negative for AAL binding (Kramer et al., 2002; Raetz and Whitfield, 2002). The main glycans exposed on the cell wall of gram-negative bacteria consist of the oligosaccharide core of lipopolysaccharides (LPS) and capsular polysaccharides, which both induce strong antibody responses (Comstock and Kasper, 2006). To determine the nature of fucosylated oligosaccharides on *Bacteroides* surfaces, we examined AAL reactivity for bacterial proteins and for LPS. Lectin blots confirmed the presence of fucosylated glycoproteins on *B. thetaiotaomicron, B. vulgatus, B. intestinalis, B. acidifaciens*, and *B. stercoris* (Figure 5B). By contrast, AAL only bound to LPS of *B. intestinalis* (Figure 5C). The fucosylated LPS from *E. coli* O127:B8 (Widmalm and Leontein, 1993) was included as a positive control to confirm the specificity of AAL toward fucose (Figure 5C). In contrast, in accordance with flow cytometry data (Figure 5A), AAL did neither react with lysate nor with non-fucosylated LPS from *E. coli* K12 (Figures 5B,C) (Kramer et al., 2002; Raetz and Whitfield, 2002).

**FIGURE 6:**
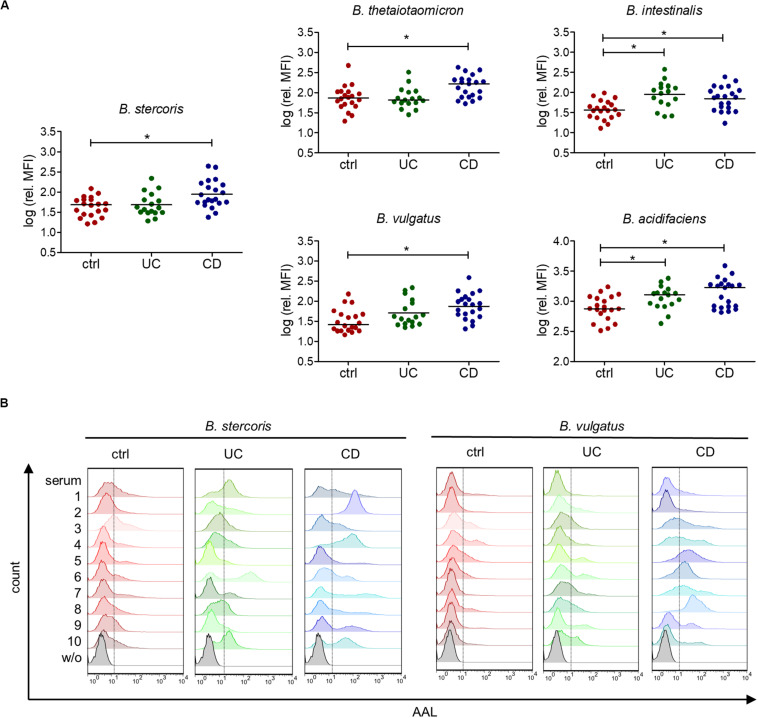
Increased serum IgG reactivity to commensal bacteria of the genus *Bacteroides* in IBD patients. **(A)** Mean fluorescence intensity (MFI) of the serum IgG reactivity to cultured *Bacteroides* species normalized to the IgG concentration of each serum, determined by flow cytometry (*n* = 20 for ctrl, *n* = 17 for UC, *n* = 21 for CD). **(B)** Histograms of 10 samples per group, showing IgG reactivity to indicated *Bacteroides* species, measured by flow cytometry as indicated in **(A)**. *p-value ≥ 0.05, determined using one-way ANOVA and Bonferroni’s *post hoc* test with selected pairs for ctrl vs UC and ctrl vs CD.

Further, we confirmed the increased reactivity of IgG from CD patients toward *B. stercoris* and other *Bacteroides* species in the serum of CD patients by flow cytometry (Figure 6A). Increased reactivity toward *B. intestinalis* and *B. acidifaciens* was also noted for IgG from UC patients. The IgG binding profiles of individual sera toward *B. stercoris* and *B. vulgatus* clearly showed the inter-individual variability in the recognition of these bacteria by serum IgG and underlined the strong reactivity of CD sera against these *Bacteroides* species (Figure 6B). Overall, our study demonstrated an increased serum antibody reactivity to fucosylated oligosaccharides and to fucose-carrying members of the genus *Bacteroides* in CD.

## Discussion

The investigation of carbohydrate-specific antibodies in IBD patients revealed an increased antibody response to oligosaccharides in CD patients, which was mainly reflected by a higher reactivity to fucosylated oligosaccharides. Previous studies reported an increased serum IgG reactivity to intestinal commensal bacteria in CD (Adams et al., 2008; Haas et al., 2011; Harmsen et al., 2012). Our work indicates that this increased reactivity to bacteria also leads to changes in the specificity of serum IgG and IgM to oligosaccharide structures. The changes in antibody specificity are likely the result of the intestinal dysbiosis that prevails in UC and CD (Nishida et al., 2018). In addition, we observed a decreased diversity in the repertoire of anti-bacterial IgG in CD patients compared with healthy controls and UC patients. The lower diversity of anti-bacterial IgG likely reflects the decreased microbial diversity reported in CD patients (Andoh et al., 2012; Quince et al., 2015; Gong et al., 2016). We observed an increased reactivity of antibodies to fucosylated oligosaccharides and commensal bacteria in the sera of CD patients, but not of UC patients. A reason for the stronger response recorded in CD may be related to the increased intestinal permeability that mainly occurs in CD patients, which induces stronger antibody response to intestinal microbes (Oriishi et al., 1995; Gerova et al., 2011). Interestingly, intestinal dysbiosis is known for UC and CD patients, but the changes in microbial composition (Machiels et al., 2014; Pascal et al., 2017) and the way how the host immune system respond to dysbiosis appear to differentially influence the emergence of carbohydrate-specific antibodies.

Increased serum IgG reactivity to fucosylated oligosaccharides possibly reflects an increase of intestinal microbes expressing fucosylated surface glycans. Our study showed that several *Bacteroides* species carry fucosylated surface glycans and may contribute to the increased IgG reactivity to fucosylated oligosaccharides in CD. Typical for gram-negative bacteria, *Bacteroides* species express a broad diversity of glycoconjugates including LPS, capsular polysaccharides and outer membrane glycoproteins (Poxton and Edmond, 1995; Jacobson et al., 2018), some of them being fucosylated (Kasper et al., 1983; Meisel-Mikolajczyk et al., 1989). Several *Bacteroides* are able to cleave terminal fucose residues and to internalize and incorporate them into their own glycans (Hooper et al., 1999; Coyne et al., 2005; Comstock and Kasper, 2006). Fucosylation of surface glycans also confers a survival advantage to *B. fragilis* in the host intestine (Coyne et al., 2005). While fucose is common on intestinal bacterial surfaces, the sialic acid N-acetylneuraminic acid is less frequent and usually associated with pathogens such as *E. coli* and *C. jejuni* (Vimr et al., 2004). Surface sialic acid often increases the virulence of bacteria by enabling the bacteria to evade immune recognition, given that sialic acid is a dominant component of host glycoconjugates that acts as a self-associated molecular pattern (Varki, 2011). The immune-dampening effect of sialic acid likely also contributes to the low amount of IgG reacting against sialylated oligosaccharides in CD sera.

In addition to changes in bacterial fucosylation, mucosal glycosylation also undergoes remodeling during intestinal inflammation. Inflammatory cytokines and tissue injury increase fucosylation in the small intestine as shown by the up-regulation of the *FUT2* fucosyltransferase gene (Umesaki and Ohara, 1989). Interestingly, intestinal bacteria also enhance fucosylation in the small intestine. This stimulatory effect appears to be specific to bacterial taxa, such as segmented filamentous bacteria and *B. thetaiotaomicron* (Umesaki et al., 1995; Bry et al., 1996), whereas others, such as Lactobacilli remain non-stimulatory (Goto et al., 2014).

This study focused on the specificity of carbohydrate-specific antibodies and their reactivity toward intestinal bacteria in IBD. The impact of serum antibodies recognizing fucosylated oligosaccharides in host tissues has not been addressed. Given the structural similarities between some bacterial glycans and host mucosal glycans (Marcobal et al., 2011; Koropatkin et al., 2012), antibodies to bacterial oligosaccharides may cross-react with host glycoproteins. Elevated levels of specific anti-carbohydrate antibodies have been linked to IBD and autoimmune diseases, including Guillain-Barré syndrome, multiple sclerosis, systemic lupus erythematosus and diabetes type 1 (Gillard et al., 1989; Schwerer, 2002; Schwarz et al., 2006; Dai et al., 2009).

Investigation of the repertoires of carbohydrate-specific antibodies in other diseases than IBD may reveal new serum markers and could help to identify microbial taxa linked to inflammatory and auto-immune diseases.

## Materials and Methods

### Materials

Anti-human IgG DyLight488 was obtained from Abcam (Cambridge, MA, United States). Goat anti-human IgG Alexa488 and goat anti-human IgM Alexa647 were purchased from Jackson ImmunoResearch Laboratories (West Grove, PA, United States). Biotinylated and fluorescein labeled AAL was bought from Vector Laboratories (Burlingame, CA, United States). LPS from *E. coli* O127:B8 was purchased from Sigma-Aldrich (St. Louis, MO, United States). Streptavidin-HRP was bought from BD Pharmingen (Franklin Lakes, NJ, United States). *Bacteroides vulgatus* ATCC 8482 (DSM 1447), *Bacteroides intestinalis* JCM 13265 (DSM 17393), *Bacteroides thetaiotaomicron* ATCC 29148 (DSM 2079), *Bacteroides acidifaciens* JCM 10556 (DSM 15896) and *Bacteroides stercoris* ATCC 43183 (DSM 19555) were obtained from the German Collection of Microorganisms and Cell Cultures (DSMZ, Braunschweig, Germany). *E. coli* K12 DH5-α was bought from Thermo Fisher Scientific (Waltham, MA, United States).

### Patients and Serum Samples

Blood serum samples from 40 IBD patients were provided by the Swiss IBD Cohort^1^. The study of the serum samples was approved by the Cantonal Ethics Committee of Zurich (KEK-ZH Nr. 2007-1316). Venous blood samples (10 mL) from healthy volunteers (without a history of chronic gastrointestinal or autoimmune diseases) were collected into non-treated BD vacutainer blood collection tubes (BD Biosciences, Franklin Lakes, NJ, United States). Clotted blood was centrifuged at 3,000 × *g* for 15 min at 4°C and the supernatant serum was frozen at −20°C. For all serum samples, IgG and IgM concentrations were determined by ELISA Kits (IgG human ELISA Kit from Abnova, IgM human ELISA Kit from Abnova (Taipeh, China) according to the manufacturer’s protocol, using a serum dilution of 1:2.000.000 for IgG and 1:60.000 for IgM.

### Oligosaccharide Arrays

The human milk shotgun glycan microarray (version 223) has been previously described (Yu et al., 2014) and was provided by National Center for Functional Glycomics (NCFG), BIDMC, Harvard University. The composition of glycans on the array is listed in Supplementary Table S1. Individual slides were loaded with 8-well format ProPlate Chambers (Grace Bio-Labs, OR, United States). After hydrating with TSM-T [20 mM Tris-HCl pH 7.4, 150 mM NaCl, 2 mM CaCl2, 2 mM MgCl2 (TSM) with 0.05% Tween], arrays were incubated with serum diluted to 500 μg/mL IgG in binding buffer (TSM-T with 1% BSA) for 1 h at room temperature, washed (4 × TSMT-T, 4 × TSM) and incubated for 1 h at room temperature with 5 μg/mL Alexa488-labeled goat anti-human IgG in binding buffer. After washing as described above, the slides were incubated for 1 h at room temperature with 5 μg/mL Alexa647 goat anti-human IgM and washed as described with a final wash of four times with deionized water. After spin-drying, arrays were scanned at 647and 488 nm using Innoscan 1100 AL (Innopsys, Carbonne, France). A mask representing the layout of the array was fit to each image using MAPIX analysis software version 8.5.0 (Innopsys, Carbonne, France). Background fluorescence was determined as the mean fluorescence signal from areas of a circle of 330 μm diameter outside the actual spot of 110 μm diameter. Background signal was subtracted, the mean of four replicates was calculated and negative values were set to 1. For further analysis log_2_ (RFU) was used. Microarray production and analyses were performed according to the MIRAGE guidelines (Liu et al., 2017) (Supplementary Data).

### Bacterial Cultures

*Bacteroides* species were cultivated anaerobically in rubber-sealed hungate tubes at 37°C in Peptone Yeast Glucose medium (DSMZ, medium no. 104 with 1.25 mg/mL glucose). *E. coli K 12* was cultivated aerobically in Luria-Bertani medium at 37°C. Cells were harvested at OD = 1.0 in aliquots of 0.5 mL, pelleted by centrifugation at 13.000 × *g* for 1 min, washed with phosphate-buffered saline (PBS) and either stored at −20°C or fixed with 2% paraformaldehyde in PBS for 15 min. 10 mL of complex bacterial cultures from a continuous *in vitro* intestinal fermentation system (Poeker et al., 2018) inoculated with fecal microbiota of a healthy human adult were directly transferred from the fermenter into a sterile, CO_2_-flushed hungate tube, which was kept on ice. The culture was centrifuged at 13,000 × *g* for 1 min to receive bacterial pellets. Bacteria were fixed using 2% paraformaldehyde in PBS for 15 min followed by a washing step with PBS.

### Flow Cytometry

Fixed bacteria were treated with 10 μg/mL fluorescein labeled AAL in PBS or diluted serum (1:100) in PBS for 1 h at room temperature and washed twice using PBS. Serum-treated bacteria were further stained with anti-human IgG DyLight488 for 1 h at room temperature and washed twice with PBS. Fluorescence was recorded by a FACSCanto II Flow cytometer (BD Biosciences, Franklin Lakes, NJ, United States). AAL positive and IgG positive bacteria were sorted using a FACSAria III Cell Sorter (BD Bioscience, Franklin Lakes, NJ, United States). The analysis was performed using the FlowJo software (BD Bioscience, Franklin Lakes, NJ, United States).

### 16S rRNA Sequencing

Bacterial pellets were treated with Proteinase K (2% V/V of 20 mg/mL stock, ThermoScientific, Waltham, MA, United States) in lysis buffer (50 mM KCl, 10 mM Tris Base, 0.45% Non-idet P-40, 0.45% Tween 20) for 45 min at 50°C. After the inactivation of Proteinase K at 95°C for 10 min, the addition of two volumes of PBS and centrifugation at 13.000 × *g* for 1 min, the supernatant, containing DNA, was stored at −20°C. The V3,V4 region was amplified by PCR using 1 μL DNA template, 200 nM primers 341Fnex0-3 and 802Rnex0-3 (Table 2), Taq DNA polymerase from Thermus aquaticus (Sigma-Aldrich, St. Louis, MO, United States) and 100 μM dNTP (ThermoScientific, Waltham, MA, United States) with the following conditions: initial denaturation at 95°C for 5 min, 30 cycles at 98°C for 20 s, 56°C for 15 s, and 72°C for 15 s, and a final step at 72°C for 2 min. PCR products were cleaned using magnetic SPRI beads [self-made by Genetic Diversity Center, ETH Zurich, Switzerland (DeAngelis et al., 1995)]. In brief, 0.8× SPRI beads were added to each reaction mix and incubated for 5 min at room temperature. The supernatant was discarded, beads were washed twice using 80% ethanol and DNA was eluted from air-dried beads in 10 mM Tris pH 8.5. A second PCR was performed using primers with Illumina adaptors (Nextera XT Index Kit v2, Illumina, San Diego, CA, United States) according to the manufacturer’s manual. PCR products were cleaned using magnetic SPRI beads as described above. DNA concentration of each sample was determined using the dsDNA HS Assay Kit (ThermoScientific, Waltham, MA, United States) according to the manufacturer’s protocol. All samples were normalized and pooled. Amplicons were sequenced using a paired-end 600 cycles (PE300) Illumina-MiSeq system (Illumina, San Diego, CA, United States) and quality controlled with FastQC (Andrews, 2010) and MultiQC (Ewels et al., 2016). The raw reads were end-trimmed using USEARCH (Edgar, 2010) and pairs were merged using FLASH (Magoc and Salzberg, 2011). Primer sequences were trimmed (Edgar, 2010) and reads were quality controlled using PRINSEQ (Schmieder and Edwards, 2011). UPARSE and UNOISE3 were used to cluster the data into OTUs, zero-radius OTUs and obtain count tables (Edgar, 2010). Taxonomic predictions were assigned using Sintax (Edgar, 2016) with the EzBioCloud 16S database (Yoon et al., 2017). For each OTU within each sample, the percentage of total bacteria was calculated. Fold enrichment of taxa was quantified as proportion of the corresponding unsorted bacterial sample.

**TABLE 2:** Primer for 16S sequencing of V3V4 region.

name	Nextera adapter	linker	shift	primer site (5′–3′)
341F_nex0	TCGTCGGCAGCGTC	AGATGTGTATAAGAGACAG	T	CCTACGGGNGGCWGCAG
341F_nex1	TCGTCGGCAGCGTC	AGATGTGTATAAGAGACAG	NT	CCTACGGGNGGCWGCAG
341F_nex2	TCGTCGGCAGCGTC	AGATGTGTATAAGAGACAG	NNT	CCTACGGGNGGCWGCAG
341F_nex3	TCGTCGGCAGCGTC	AGATGTGTATAAGAGACAG	NNNT	CCTACGGGNGGCWGCAG
802R_nex0	GTCTCGTGGGCTCGG	AGATGTGTATAAGAGACAG	T	TACNVGGGTATCTAATCC
802R_nex1	GTCTCGTGGGCTCGG	AGATGTGTATAAGAGACAG	NT	TACNVGGGTATCTAATCC
802R_nex2	GTCTCGTGGGCTCGG	AGATGTGTATAAGAGACAG	NNT	TACNVGGGTATCTAATCC
802R_nex3	GTCTCGTGGGCTCGG	AGATGTGTATAAGAGACAG	NNNT	TACNVGGGTATCTAATCC

### Quantitative PCR

*Bacteroides* spp. and *B. stercoris* were quantified as proportion of total bacterial 16S rRNA amplicons by real-time PCR using the KAPA SYBR FAST qPCR Master Mix Kit (Kapa Biosystems, Wilmington, MA, United States). Primer pairs for total bacteria (515F: 5′-GTGCCAGCMGCCGCGGTAA-3′; 805R: 5′-GACTACCAGGGTATCTAAT-3′) (Frank et al., 2007), *Bacteroides* spp. (forward: 5′-AAGGTCCCCCACATTGG-3′; reverse: 5′-GAGCCGCAAACTTTCACAA-3′) (Manz et al., 1996; Franks et al., 1998; Hermann-Bank et al., 2013) and *B. stercoris* (forward: 5′ GCTTGCTTTGATGGATGGC; reverse: 5′ CATGCGGGAAAACTATGCC) (Tong et al., 2011) were described previously. PCR conditions were initial denaturation at 95°C for 3 min, 50 cycles at 95°C for 10 s and 60°C (62°C) for 30 s, and a final dissociation curve with a gradient from 60°C (62°C) to 95°C, where 60°C was used as annealing temperature for *Bacteroides* spp. primers and 62°C for *B. stercoris* primers. Each sample was additionally amplified with primers for total bacteria on the same 96-well plate. The expression relative to total bacterial 16S rRNA amplicons was calculated using the 2^–Δ^ ^*Ct*^ method (Schmittgen and Livak, 2008).

### Lectin Blots and SDS-PAGE

Bacterial pellets were resuspended in 200 μL SDS buffer (2% β-mercaptoethanol, 2% SDS, 10% glycerol in 50 mM Tris-HCl, bromphenol blue, pH 6.8), boiled at 99°C for 15 min and cell lysates were stored at −20°C. LPS was extracted by hot aqueous-phenol extraction (Davis and Goldberg, 2012) and stored at −20°C. For lectin blots, either 7.5 μL bacterial cell lysates or 5 μL LPS, diluted 1:1 in 2^∗^SDS buffer, were run on 14% acrylamide gels at 80 V for 2 h. Gels with cell lysates were stained with colloidal Coomassie (Dyballa and Metzger, 2009) and gels with LPS were stained by silver staining (Blum et al., 1987; Fomsgaard et al., 1990). In brief, gels were incubated in an oxidation solution consisting of 40% ethanol, 5% acetic acid, 0.7% periodic acid for 20 min, washed twice in 30% ethanol, 1× deionized water, 20 min each and incubated in 0.02% sodium thiosulfate for 1 min. Gels were washed three times with deionized water and stained with 0.1% silver nitrate for 20 min at 4°C. Washed gels were developed using 3% sodium carbonate and 0.05% formaldehyde 37% until clear bands were visible. Development was stopped by 5% acetic acid for 5 min and gels were scanned (CanoScan 9000F Mark II scanner, Canon, Tokyo, Japan). For lectin blots, SDS-PAGE gels were transferred to PVDF membranes at 250 mA for 1 h. Membranes were blocked ON at 4°C with 1% BSA in PBS-T (PBS with 0.1% Tween20) and incubated with 10 μg/mL biotinylated AAL or with 10 μg/mL biotinylated AAL and 200 mM L-fucose in PBS-T for 1 h at room temperature, after a pre-incubation of AAL and L-fucose for 30 min. Membranes were washed four times with PBS-T for 10 min, incubated with Streptavidin-HRP (1:1000) for 1 h at room temperature. After another washing step, identical to the one described above, membranes were detected using SuperSignal^®^ West Pico chemiluminescence substrate (Thermo Fisher Scientific, Waltham, MA, United States) and a Fujifilm LAS-4000 luminescence image analyzer (GE Healthcare, Chicago, IL, United States).

### Statistics

Data are shown as median values and standard deviation. Statistical analysis was performed using GraphPad Prism (GraphPad software, San Diego, CA, United States). For comparison of two groups (unsorted bacteria vs IgG+, unsorted bacteria vs AAL+), a student’s *t*-test was used. For comparison of multiple groups (ctrl, UC, CD), one-way ANOVA and Bonferroni’s *post hoc* test for Gauss distributed samples or Kruskal-Wallis test and Dunn’s *post hoc* test for non-Gauss distributed samples with selected pairs of ctrl vs CD and ctrl vs UC were applied as indicated in the Figure legends. Results with *p*-value ≤ 0.05 were considered significantly different. Pearson’s correlation analysis was performed using GraphPad Prism. To compare the IgG reactivity to single oligosaccharides for patients and control groupsa software tool run on R (version V3.6.1) using the “limma” package (Ritchie et al., 2015), fitting a linear model for each feature and testing differential reactivity, with a modified *t*-test was used. Unsupervised hierarchical cluster analysis is based on a matrix with the correlation coefficients of all sample pairs. Clustering and visualization were performed using the “complete-linkage” method in R (version V3.6.1) and the R package pheatmap.

## Data Availability Statement

The datasets presented in this study can be found in online repositories. The names of the repository/repositories and accession number(s) can be found at: https://www.ebi.ac.uk/ena, PRJEB37080.

## Ethics Statement

The studies involving human participants were reviewed and approved by Cantonal Ethics Committee of Zurich (KEK-ZH Nr. 2007-1316). The patients/participants provided their written informed consent to participate in this study.

## Author Contributions

TH designed the study and secured the funding. KK performed and analyzed the experiments. YL and KK performed the array experiments and analysis with advice from DS. LO assisted in the analysis of array data and gave advice on the statistical analysis. KK and TH wrote the manuscript. DS and YL revised the manuscript. All authors contributed to the article and approved the submitted version.

## Conflict of Interest

The authors declare that the research was conducted in the absence of any commercial or financial relationships that could be construed as a potential conflict of interest.

## Footnotes

1 http://www.ibdcohort.ch/
