# Supplementary material for: Increased Antibody Response to Fucosylated Oligosaccharides and Fucose-Carrying Bacteroides Species in Crohn’s Disease
Source: Front Microbiol. 2020 Jul 14;11:1553. doi: 10.3389/fmicb.2020.01553 (PMC7381230; doi:10.3389/fmicb.2020.01553)
Supplement: Supplementary file 1 [file Data_Sheet_1.PDF]

Supplementary Material

Supplementary Tables

Supplementary Table S1 (see additional Excel file)

Supplementary Table S2: Correlation of IgM and IgG reactivities to oligosaccharides.

	control		CD		UC	
sample	r	P < 0.0001	r	P < 0.0001	r	P < 0.0001
1	0.9675	y	0.8881	y	0.6754	y
2	0.9411	y	0.8102	y	0.9162	y
3	0.9525	y	0.9330	y	0.8343	y
4	0.8764	y	0.9822	y	0.8343	y
5	0.8775	y	0.8885	y	0.9318	y
6	0.9174	y	0.9507	y	0.7705	y
7	0.6697	y	0.9285	y	0.9216	y
8	0.3875	y	0.9233	y	0.9296	y
9	0.9349	y	0.7866	y	0.8242	y
10	0.8356	y	0.9312	y	0.9385	y
11	0.9018	y	0.8657	y	0.8786	y
12	0.9124	y	0.9330	y	0.8545	y
13	0.6734	y	0.8900	y	0.9847	y
14	0.9168	y	0.9593	y	0.9197	y
15	0.7644	y	0.9537	y	0.9549	y
16	0.7375	y	0.9441	y	0.9000	y
17	0.8910	y	0.9358	y		
18	0.7982	y	0.7652	y		
19	0.9161	y	0.9364	y		
20	0.8311	y	0.9140	y		
21			0.9806	y		
22			0.9270	y		
23			0.8883	y		

r = Pearson’s correlation coefficient
p = P value
y = yes

Supplementary Figures

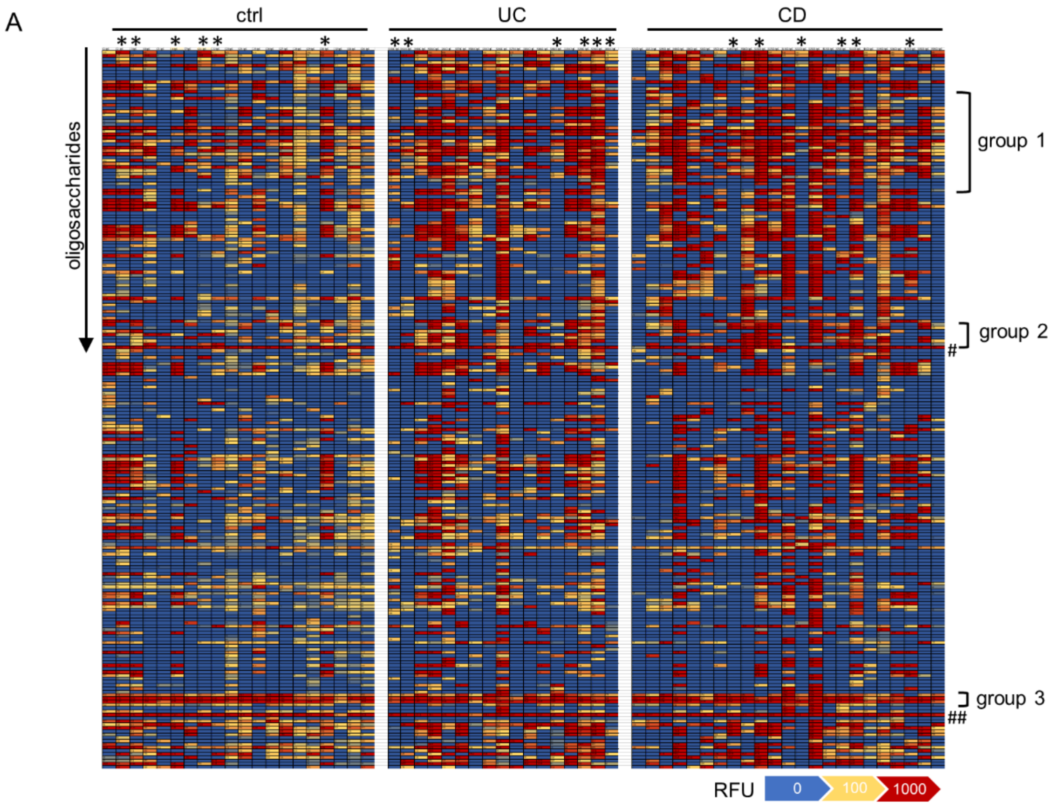

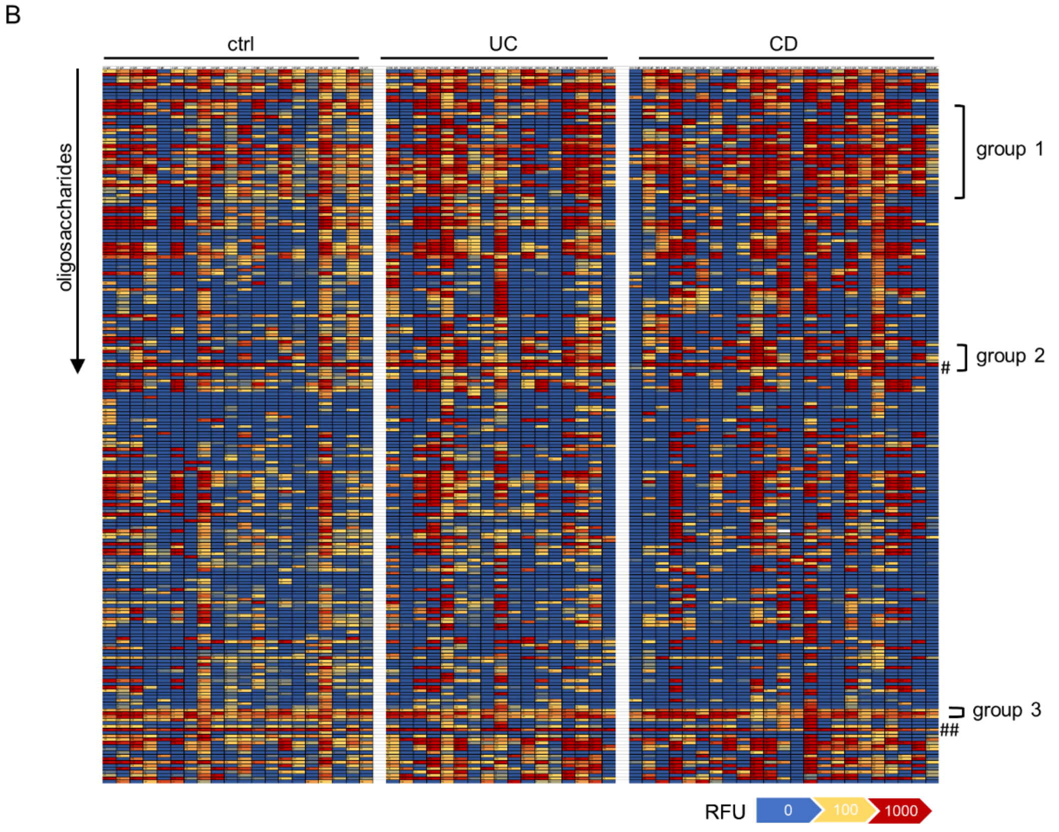

Supplementary Figure S1: Increased serum IgG and IgM reactivity to oligosaccharides in CD. Heatmap of the (A) IgG reactivity and (B) IgM to 220 different oligosaccharide structures for blood serum from healthy controls (n=20), UC (n=17) and CD (n=23) patients, based on the fluorescence intensities (RFU) measured by glycan array analysis. Prominent oligosaccharides or groups of oligosaccharides are labeled with # or a group number. Samples used for Fig. 3B are indicated by asterisks.

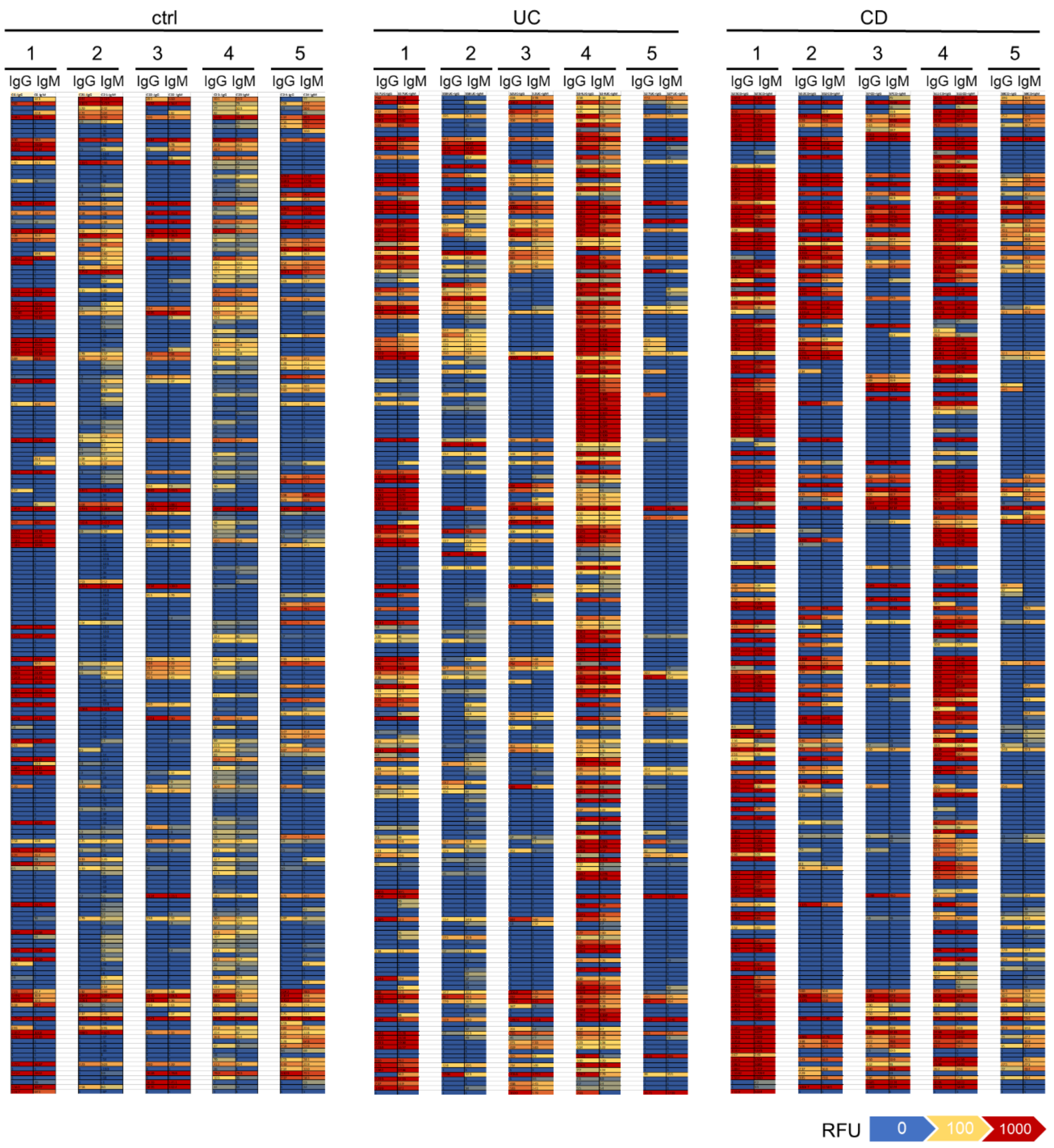

Supplementary Figure S2: Oligosaccharide-specific IgM and IgG profiles of each person are comparable. Heatmaps of the IgG and IgM reactivities for five samples per group (ctrl, UC, CD).

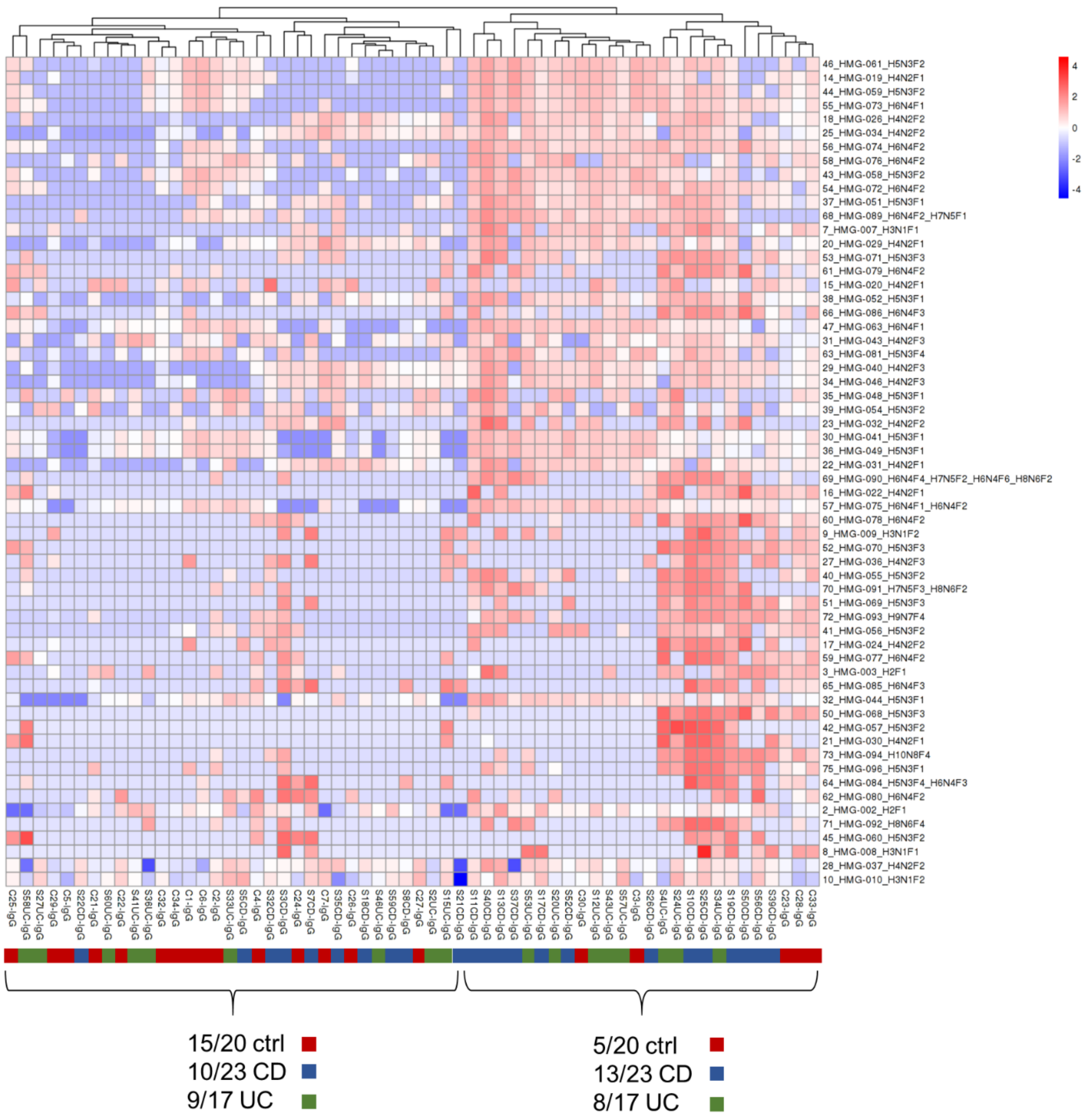

Supplementary Figure S3: High IgG reactivity to fucosylated oligosaccharides is frequent among CD samples but rare in control samples. Hierarchical cluster analysis of control (ctrl) (n=20), UC (n=17) and CD (n=23) samples for the IgG reactivity to fucosylated non-sialylated oligosaccharides determined by glycan array analysis. For each group (ctrl, UC, CD), the number of samples out of the total number of samples within a cluster is indicated for the biggest two clusters.

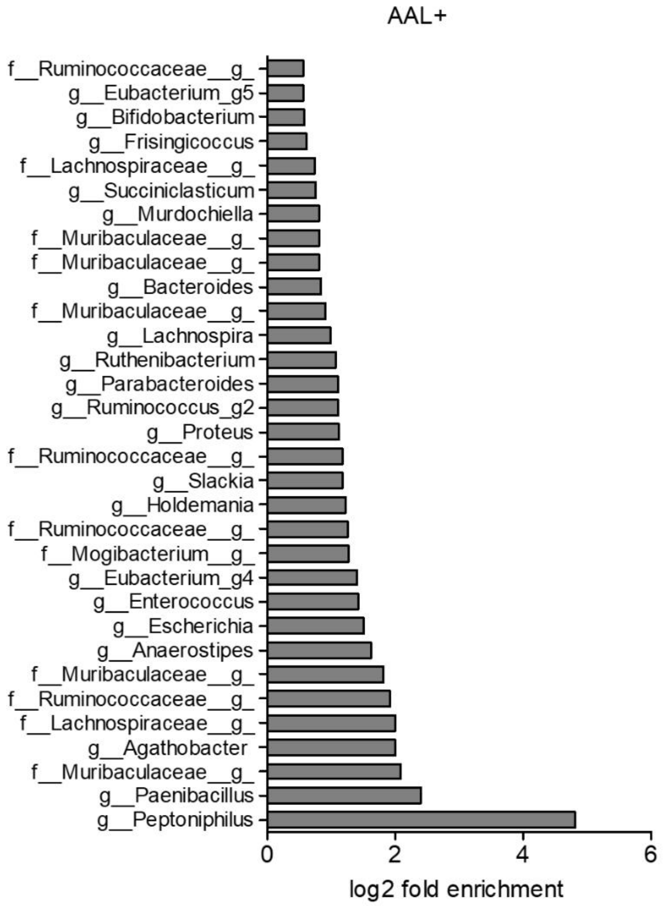

Supplementary Figure S4: Bacteria recognized by the fucose-binding lectin AAL. Enrichment of AAL-bound bacteria compared with unsorted bacteria with taxonomic assignments down to the family or genus level.

ECGC Supplemental glycan microarray document based on MIRAGE Guidelines (doi:10.3762/mirage.3)

Array Name from Vendor: HUMAN MILK GLYCAN 223

Source: Glycomics Core/BIDMC/Harvard Medical School

Array name assigned by ECGC: HMO223

Date received: 7/18/2017

Slide#: 25

Labeled Index: In-printed Barcode

<table>
<tr><th colspan="2">Classification</th></tr>
<tr><td colspan="2">1. Sample: Glycan Binding Sample</td></tr>
<tr><td>Description of Sample</td><td>human blood serum</td></tr>
<tr><td>Sample modifications</td><td>diluted to 500 µg/ml IgG</td></tr>
<tr><td>Assay protocol</td><td>After hydrating with TSM-T, arrays were incubated with serum diluted to 500 µg/ml IgG in binding buffer (TSM-T with 1% BSA) for 1 h at room temperature, washed (4 x TSMT-T, 4 x TSM) and incubated for 1 h at room temperature with 5 µg/ml Alexa488-labeled goat anti-human IgG in binding buffer. After washing as described above, the slides were incubated for 1 h at room temperature with 5 µg/ml Alexa647 goat anti-human IgM and washed as described with a final wash of four times with deionized water and spin-dried.</td></tr>
<tr><td colspan="2">2. Glycan Library</td></tr>
<tr><td>Glycan description for defined glycans</td><td>Defined glycans were purchased from Elicityl.
3'-Fucosyllactose / 3'-FL (GLY060)
Lacto-N-tetraose / LNT (GLY010)
Lacto-N-neotetraose / LNnT (GLY021)
Blood Group H antigen pentaose type I / Lacto-N-fucopentaose I / LNFP I (GLY033-1)
Lewis Y (LeY) pentaose (GLY052)
Lacto-N-fucopentaose III / LNFP III
Lacto-N-neofucopentaose / LNnFP V / LNFP IV (GLY061)
Lacto-N-fucopentaose V / LNFP V (GLY062)
Sialyl-lacto-N-tetraose a / LSTa (GLY081)
Sialyl-lacto-N-tetraose b / LSTb
Sialyl-lacto-N-tetraose a / LSTc
Sialylated tetraose type I / Sialyl Lewisa precursor (GLY080)
Lewis X (LeX) tetraose (GLY050)</td></tr>
<tr><td>Glycan description for undefined glycans</td><td>Human milk glycans were isolated from skimmed milk.</td></tr>
</table>

Glycan modifications	Glycans were conjugated with AEAB and separated by HPLC. See Yu *et al*. 2012 and Yu *et al*. 2014 for shotgun glycan preparation schema.
3. Printing Surface; e.g., Microarray Slide	
Description of surface	Schott type H slides
Manufacturer	SCHOTT North America, Inc., Elmsford, NY
Custom preparation of surface	No
Non-covalent Immobilization	Yes
Covalent Immobilization	No
4. Arrayer (Printer)	
Description of Arrayer	Scienion sciFLEXARRAYER S11 Microarray Printer
Dispensing mechanism	TGL glycans were dispensed at ~333 picoliter per spot to produce ~ 110 micron diameter spots.
Glycan deposition	TGL glycans were dispensed at various concentration (see appendix I) in sodium phosphate buffer (100 mM, pH 8.5).
Printing conditions	Relative humidity was maintained at 60%. Slides were blocked with 50 mM Ethanolamine in 100 mM Sodium tetraborate buffer (pH 8.5). Printed slides were stored at -20°C before use.
5. Glycan Microarray with "Map"	
Array layout	Glycan microarray slides were printed with 8 (1 x 8) subarrays. Each sample was printed in 4 replicates.<br>Incubation chamber: 8-well format ProPlate Chamber (Grace Bio-Labs, OR, USA).
Glycan identification and quality control	Visual inspection of printed spots
6. Detector and Data Processing	
Scanning hardware	InnoScan 1100 AL scaner (Innopsys, Carbonne, France)
Scanner settings	Scanning resolution: 5 μm/pixel;<br>Laser channel 635 nm: PMT gain: 35, Power: low;<br>Laser channel 488 nm: PMT gain: 80, Power: high.
Image analysis software	Mapix version 8.2.7. (Innopsys, Carbonne, France)
Data processing	The gpr files were processed with an in-house workflow using excel macro. No particular normalization method was used. The background signal was subtracted, the mean of four replicates was calculated (RFU) and negative values were set to 1.
7. Glycan Microarray Data Presentation	
Data presentation	Data is presented as mean RFU or log (RFU) of single glycans or subgroups of glycans. Statistical analysis was performed using GraphPadPrism version 5.03 (GraphPad software, San Diego, CA) for one-Way ANOVA and Bonferroni's multiple comparison test for Gauss distributed samples or Kruskal-Wallis test and Dunn's post hoc test for non-Gauss distributed samples. Pearson's correlation analysis was performed using GraphPad Prism version 5.03. To compare the IgG reactivity to single oligosaccharides for patients' and control groups, a software tool run on R (version V3.6.1) using the "limma" package (Ritchie *et al*. 2015) was used.
8. Interpretation and Conclusion from Microarray Data	
Data interpretation	see Results and Discussion
Conclusions	see Results and Discussion

References:

Yu, Y, Mishra, S, Song X, Lasanajak Y, Bradley KC, Tappert MM, *et al*. Functional glycomic analysis of human milk glycans reveals the presence of virus receptors and embryonic stem cell biomarkers. Journal of Biological Chemistry. 2012; 287(53):44784-44799. doi:10.1074/jbc.M112.425819.

Yu Y, Lasanajak Y, Song X, Hu L, Ramani S, Mickum ML, *et al*. Human milk contains novel glycans that are potential decoy receptors for neonatal rotaviruses. Molecular and Cellular Proteomics. 2014; 13(11):2944-2960. doi: 10.1074/mcp.M114.039875.

Ritchie ME, Phipson B, Wu D, Hu Y, Law CW, Shi W, *et al.* limma powers differential expression analyses for RNA-sequencing and microarray studies. Nucleic acids research. 2015;43(7):e47. doi: 10.1093/nar/gkv007.
